# *TCF12* is mutated in anaplastic oligodendroglioma

**DOI:** 10.1038/ncomms8207

**Published:** 2015-06-12

**Authors:** Karim Labreche, Iva Simeonova, Aurélie Kamoun, Vincent Gleize, Daniel Chubb, Eric Letouzé, Yasser Riazalhosseini, Sara E. Dobbins, Nabila Elarouci, Francois Ducray, Aurélien de Reyniès, Diana Zelenika, Christopher P. Wardell, Mathew Frampton, Olivier Saulnier, Tomi Pastinen, Sabrina Hallout, Dominique Figarella-Branger, Caroline Dehais, Ahmed Idbaih, Karima Mokhtari, Jean-Yves Delattre, Emmanuelle Huillard, G. Mark Lathrop, Marc Sanson, Richard S. Houlston, POLA Network, Clovis Adam, Marie Andraud, Marie-Hélène Aubriot-Lorton, Luc Bauchet, Patrick Beauchesne, Claire Blechet, Mario Campone, Antoine Carpentier, Catherine Carpentier, Ioana Carpiuc, Marie-Pierre Chenard, Danchristian Chiforeanu, Olivier Chinot, Elisabeth Cohen-Moyal, Philippe Colin, Phong Dam-Hieu, Christine Desenclos, Nicolas Desse, Frederic Dhermain, Marie-Danièle Diebold, Sandrine Eimer, Thierry Faillot, Mélanie Fesneau, Denys Fontaine, Stéphane Gaillard, Guillaume Gauchotte, Claude Gaultier, Francois Ghiringhelli, Joel Godard, Edouard Marcel Gueye, Jean Sebastien Guillamo, Selma Hamdi-Elouadhani, Jerome Honnorat, Jean Louis Kemeny, Toufik Khallil, Anne Jouvet, Francois Labrousse, Olivier Langlois, Annie Laquerriere, Emmanuelle Lechapt-Zalcman, Caroline Le Guérinel, Pierre-Marie Levillain, Hugues Loiseau, Delphine Loussouarn, Claude-Alain Maurage, Philippe Menei, Marie Janette Motsuo Fotso, Georges Noel, Fabrice Parker, Michel Peoc’h, Marc Polivka, Isabelle Quintin-Roué, Carole Ramirez, Damien Ricard, Pomone Richard, Valérie Rigau, Audrey Rousseau, Gwenaelle Runavot, Henri Sevestre, Marie Christine Tortel, Emmanuelle Uro-Coste, Fanny Burel-Vandenbos, Elodie Vauleon, Gabriel Viennet, Chiara Villa, Michel Wager

**Affiliations:** 1 Division of Genetics and Epidemiology, The Institute of Cancer Research, Sutton, Surrey SM2 5NG, UK; 2 Inserm, U 1127, ICM, F-75013 Paris, France; 3 CNRS, UMR 7225, ICM, F-75013 Paris, France; 4 Institut du Cerveau et de la Moelle épinière ICM, Paris 75013, France; 5 Sorbonne Universités, UPMC Université Paris 06, UMR S 1127, F-75013 Paris, France; 6 Programme Cartes d’Identité des Tumeurs (CIT), Ligue Nationale Contre Le Cancer, 75013 Paris, France; 7 Department of Human Genetics, McGill University, Montreal, Quebec, Canada H3A 0G1; 8 McGill University and Genome Quebec Innovation Centre, Montreal, Quebec, Canada H3A 0G1; 9 INSERM U1028, CNRS UMR5292, Service de Neuro-oncologie, Hopital neurologique, Hospices civils de Lyon, Lyon Neuroscience Research Center, Neuro-Oncology and Neuro-Inflammation Team, 69677 Lyon, France; 10 Centre National de Génotypage, IG/CEA, 2 rue Gaston Crémieux, CP 5721, Evry 91057, France; 11 Division of Molecular Pathology, The Institute of Cancer Research, Sutton, Surrey SM2 5NG, UK; 12 AP-HM, Hôpital de la Timone, Service d’anatomie pathologique et de neuropathologie, 13385 Marseille, France; 13 Université de la Méditerranée, Aix-Marseille, Faculté de Médecine La Timone, CRO2, UMR 911 Marseille, France; 14 AP-HP, Groupe Hospitalier Pitié-Salpêtrière, Service de neurologie 2-Mazarin, 75013 Paris, France; 15 AP-HP, Groupe Hospitalier Pitié-Salpêtrière, Laboratoire de Neuropathologie R. Escourolle, 75013 Paris, France; 16 Hôpital Bicêtre, Pathology Department, 94275 Le Kremlin-Bicêtre, France.; 17 CHU Saint-Pierre de la Réunion, Pathology Department, Saint-Pierre de la Réunion, 97410 France.; 18 CHU Dijon, Pathology Department, 21000 Dijon, France.; 19 CHU de Montpellier, Neurosurgery Department, 34295 Montpellier, France.; 20 CHU Nancy, Neuro-oncology Department, 54035 Nancy, France.; 21 CHR Orléans, Pathology Department, 45000 Orléans, France.; 22 Centre René Gauducheau, Medical Oncology Department, 44805 Saint-Herblain, France.; 23 Hôpital Avicenne, Neurology Department, 93009 Bobigny, France.; 24 Universite Pierre et Marie Curie, Centre de Recherche de l’institut du Cerveau et de la Moelle Epiniere and INSERM UMRS 975/CNR, 75013 Paris, France.; 25 Clinique des Cèdres, Medical Oncology Department, 31700 Cornebarrieu, France.; 26 CHU Strasbourg, Pathology Department, 67098 Strasbourg, France.; 27 CHU Rennes, Pathology Department, 35033 Rennes, France.; 28 Hôpital de la Timone, Assistance Publique—Hôpitaux de Marseille, Neuro-oncology Department, 13385 Marseille, France.; 29 Institut Claudius Regaud, Radiotherapy Department, 31059 Toulouse, France.; 30 Clinique de Courlancy, Radiotherapy Department, 51100 Reims, France.; 31 Hôpital de la cavale blanche, CHU Brest, Neurosurgery Department, 29609 Brest, France.; 32 Hôpital Nord, CHU Amiens, Neurosurgery Department, 80054 Amiens, France.; 33 HIA Sainte-Anne, Neurosurgery Department, 83800 Toulon, France.; 34 Institut Gustave Roussy, Radiotherapy Department, 94805 Villejuif, France.; 35 CHU Reims, Pathology Department, 51092 Reims, France.; 36 CHU de Bordeaux-GH Pellegrin, Pathology Department, 33000 Bordeaux, France.; 37 Hôpital Beaujon, Neurosurgery Department, 92110 Clichy, France.; 38 CHR Orléans, Radiotherapy Department, 45000 Orléans, France.; 39 CHU Nice, Neurosurgery Department, 06002 Nice, France.; 40 Hôpital Foch, Neurosurgery Department, 92151 Suresnes, France.; 41 CHU Nancy, Pathology Department, 54035 Nancy, France.; 42 CH Colmar, Neurology Department, 68024 Colmar, France.; 43 Centre Georges-François Leclerc, Medical Oncology, 21079 Dijon, France.; 44 Hôpital Jean Minjoz, CHU Besançon, Neurosurgery Department, 25030 Besançon, France.; 45 Hôpital Dupuytren, CHU de Limoges, Neurosurgery Department, 87042 Limoges, France.; 46 CHU de Caen, Neurology Department, 14033 Caen, France.; 47 Hôpital Lariboisière, Neurosurgery Department, 75475 Paris, France.; 48 Hospices Civils de Lyon, Hôpital Neurologique, Neuro-oncology Department, 69677 Bron, France.; 49 CHU Clermont-Ferrand, Pathology Department, 63003 Clermont-Ferrand, France.; 50 CHU Clermont-Ferrand, Neurosurgery Department, 63003 Clermont-Ferrand, France.; 51 Hospices Civils de Lyon, Hôpital Neurologique, Pathology and Neuropathology Department, 69677 Bron, France.; 52 Hôpital Dupuytren, CHU de Limoges, Pathology Department, 87042 Limoges, France.; 53 CHU Charles Nicolle, Neurosurgery Department, 76000 Rouen, France.; 54 CHU Charles Nicolle, Pathology Department, 76031 Rouen, France.; 55 CHU de Caen, Pathology Department, Caen, 14033 France.; 56 Hôpital Henri Mondor, Neurosurgery Department, 94010 Henri Mondor, France.; 57 CHU Poitiers, Neurosurgery Department, 86000 Poitiers, France.; 58 CHU de Bordeaux-GH Pellegrin, Neurosurgery Department, 33000 Bordeaux, France.; 59 CHU Nantes, Pathology Department, 44093 Nantes, France.; 60 CHU de Lille, Pathology Department, 59037 Lille, France.; 61 CHU Angers, Neurosurgery Department, 49933 Angers, France.; 62 Hôpital Nord, CHU Saint-Étienne, Neurosurgery Department, 42270 Saint-Priest en Jarez, France.; 63 Centre Paul Strauss, Radiotherapy Department, 67065 Strasbourg, France.; 64 Hôpital Bicêtre, Neurosurgery Department, 94275 Le Kremlin-Bicêtre, France.; 65 Hôpital Nord, CHU Saint-Étienne, Pathology Department, 42270 Saint-Priest en Jarez, France.; 66 Hôpital Lariboisière, Pathology Department, 75475 Paris, France.; 67 Hôpital de la cavale blanche, CHU Brest, Pathology Department, 29609 Brest, France.; 68 CHU de Lille, Neurosurgery Department, Lille, 59037 France.; 69 HIA du Val de Grâce, Neurology Department, 75230 Paris, France.; 70 Laboratoire les Feuillants, Pathology Department, 31023 Toulouse, France.; 71 CHU de Montpellier, Pathology Department, 34295 Montpellier, France.; 72 CHU Angers, Pathology Department, 49933 Angers, France.; 73 CHU Saint-Pierre de la Réunion, Neurology Department, 97410 Saint-Pierre de la Réunion, France.; 74 Hôpital Nord, CHU Amiens, Pathology Department, 80054 Amiens, France.; 75 CH Colmar, Pathology Department, 68024 Colmar, France.; 76 Hôpital Rangueil, CHU Toulouse, Pathology Department, 31059 Toulouse, France.; 77 CHU Nice, Pathology Department, 06002 Nice, France.; 78 Centre Eugène Marquis, Medical Oncology, 35042 Rennes, France.; 79 Hôpital Jean Minjoz, CHU Besançon, Pathology Department, 25030 Besançon, France.; 80 Hôpital Foch, Pathology Department, 92151 Suresnes, France.

## Abstract

Anaplastic oligodendroglioma (AO) are rare primary brain tumours that are generally incurable, with heterogeneous prognosis and few treatment targets identified. Most oligodendrogliomas have chromosomes 1p/19q co-deletion and an *IDH* mutation. Here we analysed 51 AO by whole-exome sequencing, identifying previously reported frequent somatic mutations in *CIC* and *FUBP1*. We also identified recurrent mutations in *TCF12* and in an additional series of 83 AO. Overall, 7.5% of AO are mutated for *TCF12*, which encodes an oligodendrocyte-related transcription factor. Eighty percent of *TCF12* mutations identified were in either the bHLH domain, which is important for TCF12 function as a transcription factor, or were frameshift mutations leading to TCF12 truncated for this domain. We show that these mutations compromise *TCF12* transcriptional activity and are associated with a more aggressive tumour type. Our analysis provides further insights into the unique and shared pathways driving AO.

## Abstract (web summary)

Anaplastic oligodendrogliomas are rare and incurable primary brain tumours with few treatment options. Here Labreche *et al*. perform whole-exome sequencing and identify recurring mutations in transcription factor TCF12, which are associated with aggressive tumours.

## Graphical abstract

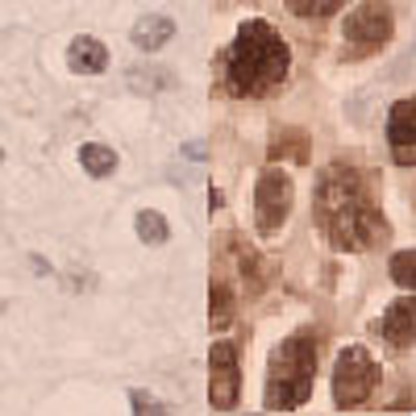

Anaplastic oligodendrogliomas (AO; World Health Organization grade III oligodendrogliomas) are rare primary malignant brain tumours with a highly variable overall prognosis. The emblematic molecular alteration in oligodendrogliomas is 1p/19q co-deletion, which is associated with a better prognosis and response to early chemotherapy with procarbazine, lomustine and vincristine^1^,^2^,^3^. Recent high-throughput sequencing approaches have identified *IDH* (*IDH1* and *IDH2*), *CIC*, *FUBP1* and *TERT* promoter mutations in oligodendroglioma (75, 50, 10 and 75%, respectively)^2^,^4^,^5^, *IDH* mutation status typically being associated with a better clinical outcome^6^. Identifying additional driver genes and altered pathways in oligodendroglioma offers the prospect of developing more effective therapies and biomarkers to predict individual patient outcome.

Here we perform whole-exome and transcriptome sequencing of AO to search for additional tumour driver mutations and pathways disrupted. In addition to previously reported recurrently mutated genes, we report the identification of somatic mutations in *TCF12* in AO. These mutations compromise *TCF12* transcriptional activity and confer a more aggressive AO phenotype.

## Results

In accordance with conventional clinical practice, we considered three molecular subtypes for our analyses: (i) *IDH*-mutated 1p/19q co-deleted (*IDH*mut-codel); (ii) *IDH*-mutated 1p/19q non-co-deleted (*IDH*mut-non-codel) and (iii) *IDH*-wild type (*IDH*wt)^7^. Assignment of *IDH*-mutated (defined by *IDH1* R132 or *IDH2* R172 mutations), 1p/19q and *TERT* promoter mutation (defined by C228T or C250T) status in tumours was determined using conventional sequencing and single-nucleotide polymorphism (SNP) array methods.

### Mutational landscape

**Figure 1:**
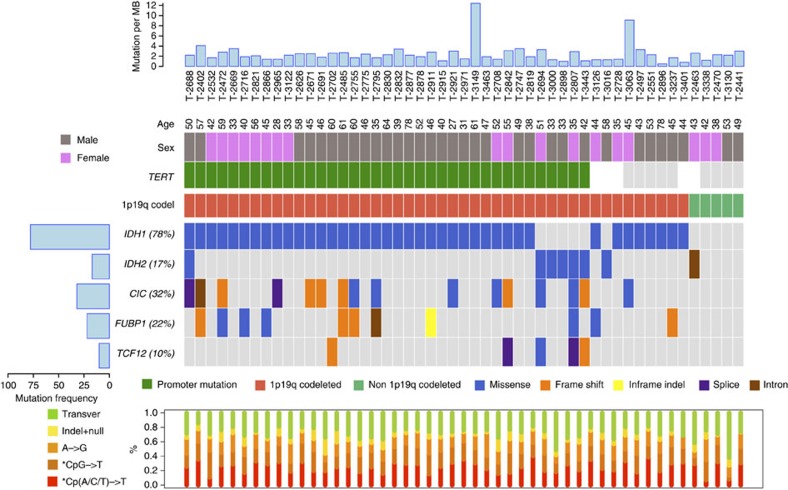
Significantly mutated genes in anaplastic oligodendroglioma by molecular subtype. Significantly mutated genes (*Q*-value<0.1) identified by exome sequencing are listed by *Q*-value. The percentage of AO samples with mutation detected by automated calling is detailed on the left. Samples are displayed as columns, with the mutation rate plotted at the top. Samples are arranged to emphasize mutual exclusivity. Mutation types are indicated in different colours (see legend). White colour indicates no information available. Also shown is the relative proportion of base-pair substitutions within mutation categories for each tumour.

We performed whole-exome sequencing of 51 AO tumours (Supplementary Data 1) and matched germline DNA, targeting 318,362 exons from 18,901 genes. The mean sequencing coverage across targeted bases was 57 × , with 80% of target bases above 20 × coverage (Supplementary Fig. 1). We identified a total of 4,733 mutations (with a mean of 37 non-silent mutations per sample) equating to a mean somatic mutation rate of 1.62 mutations per megabase (Mb) (Fig. 1). Although the tumours of two patients (3,063 and 3,149) had high rates of mutation (9.1 and 12.4, respectively), this was not reflective of tumour site (both frontal lesions as were 68% of the whole series) or treatment. Excluding these two cases the mean rate of non-silent mutations per tumour was 33±14, which is similar to the number found in most common adult brain tumours. The mutation spectrum in AO tumours was characterized by a predominance of C>T transitions, as observed in most solid cancers (Fig. 1)^8^,^9^. While few of the tumours were *IDHwt*, these did not harbour a significantly higher number of mutations compared with *IDHmut-1p/19q* co-deleted and *IDHmut-non-1p/19q* co-deleted tumours (Fig. 1). Intriguingly, one tumour (2,688) was co-mutated for *IDH1* (R132H) and *IDH2* (P162S), but exhibited no distinguishing phenotype in terms of clinicopathology or mutation rate.

We used MutSigCV version 1.4 (ref. 8) to identify genes harbouring more non-synonymous mutations than expected by chance given gene size, sequence context and mutation rate of each tumour for the three molecular subtypes, respectively. As expected, we observed frequent mutations of the tumour suppressors *FUBP1* (22%) located on 1p, and *CIC* (32%) located on 19q, which have been reported in the context of 1p/19q co-deletion (Fig. 1; Supplementary Fig. 2); these were not mutually exclusive events (Fig. 1). Also within the *IDH*mut-codel group, 37 of tumours tested carried *TERT* C228T or C250T promoter mutations (72%), none of which also carried an *ATRX* mutation, concordant with the previously reported finding that these are mutually exclusive events^2^.

In addition to the mutation of *IDH1* (78%), *IDH2* (17%), *CIC* (32%) and *FUBP1* (22%), *TCF12* was also significantly mutated (*Q*-value<0.1; Fig. 1; Supplementary Table 2). Heterozygous somatic mutations in *TCF12*, which encodes the basic helix–loop–helix (bHLH) transcription factor 12 (aliases *HEB*, *HTF4* and *ALF1*) were identified in five (1 missense, R602M; 2 splice-site, c.825+5G>T, c.1979-3_1979-delTA and 2 frameshift, E548fs*13, S682fs*14) of the 46 *IDH*-mutated 1p/19q co-deleted. Intriguingly, germline mutations of residues E548 and R602 have been previously shown to cause coronal craniosynostosis^10^.

The availability of high-quality tumour material allowed us to generate SNP array and expression data on 31 of the cases exome sequenced. In addition to co-deletion of chromosome arms 1p/19q, we identified several other recurrent genomic alterations—mainly loses of chromosomes 4 (29%), 9p (28%) and 14q (19%); Supplementary Fig. 3; Supplementary Table 1). Notably, tumours featuring mutation of Notch-pathway genes showed significant chromosome 4 loss (*P*=0.02, *χ*^2^-test). To identify fusion transcripts, we analysed RNA-sequencing (RNA-seq) data, which was available for 36 of the 51 tumours. After filtering, the only chimeric transcript identified was the predicted driver *FGFR3–TACC3* fusion, previously described in *IDH* wild-type gliomas^11^,^12^,^13^, which was seen in two of the *IDH*wt-non-1p/19q co-deleted tumours—patients 2463 and 2441; Of note was that patient 2463 carried an *IDH2* intron-5 mutation (c.679-28C>T).

### Incorporation of TCGA mutation data

To explore the mutational spectra of AO in an independent series, we made use of data generated by The Cancer Genome Atlas (TCGA) study of low-grade glioma, which provides exome sequencing data on a further 43 AO tumours. Two of these 43 tumours harboured frameshift mutations in *TCF12* (E548R and D171fs) (Supplementary Table 2). As with our series, these *TCF12* mutations were exclusive to *IDH*-1p/19q co-deleted tumours. In a combined analysis, mutations in *PI3KCA*, *NOTCH1* and *TP53* were significantly overrepresented when analysed using MutSigCV (*Q*-value<0.1; Supplementary Table 2). In addition, mutation of *ATRX* and *RBPJ* were of borderline significance.

**Figure 2:**
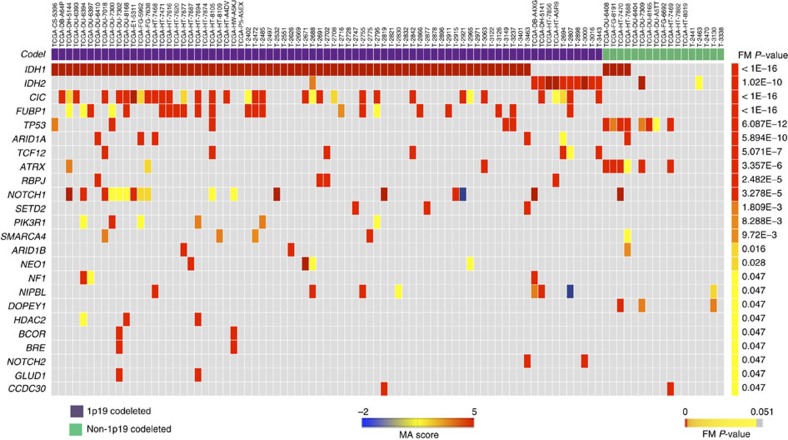
FM-biased genes and gene modules in AO identified by Oncodrive-fm using data from this study and tumours profiled by TCGA. Heatmap shows tumours in columns and genes in rows, the colour reflecting the MutationAssessor (MA) scores of somatic mutations. FM ext. qv, corrected *P* values of the FM bias analysis using the external null distribution.

A bias towards variants with functional impact (FM) is a feature of cancer drivers^14^. To increase our ability to identify cancer drivers and delineate associated oncogenic pathways for AO, we incorporated mutation data from multiple tumour types using Oncodrive-fm^14^ implemented within the IntOGen-mutations platform^15^ (Fig. 2). The most recurrently mutated genes according to MutSig were also detected by Oncodrive-fm as significantly mutated (*Q*-value<0.05). Oncodrive-fm also identified a number of other important mutated genes (that is, displaying high FM bias) including *SETD2*, *NOTCH2*, *RBPJ*, *ARID1A*, *ARID1B*, *HDAC2* and *SMARCA4* (Fig. 2).

Using all mutation results, we performed an analysis to identify pathways or gene ontologies that were significantly enriched in mutated genes. As expected, the most significantly altered pathways were linked to the tricarboxylic acid cycle and isocitrate metabolic process as a consequence of *IDH* mutation. Consistent with the other genes that were found significantly mutated by MutSigCV and Oncodrive-fm analysis, the Notch signalling pathway (*P*=1.0 × 10^−5^, binomial test), genes involved in neuron differentiation (*P*=2.0 × 10^−5^, binomial test) and genes involved in chromatin organization (*P*=0.02, binomial test) were also significantly enriched for mutations (Supplementary Data 3).

### Validation of *TCF12* in an additional series of AO

To identify additional *TCF12*-mutated AO tumours, we conducted targeted sequencing of a further 83 AO. Five tumours harboured *TCF12* mutations—G48fs*38, M260fs*5, R326S, D455fs*59 and delN606 (Supplementary Data 1). On the basis of our combined sample of 134 tumours, the mutation frequency of *TCF12* in AO is 7.5% (95% confidence interval 3.6–13.2%). No significant difference in patient survival in 1p/19q co-deleted AO was associated with *TCF12* mutation in 69 patients (Supplementary Fig. 4). While our power to demonstrate a statistically significant relationship was limited (that is, ∼40% for a hazard ratio of 2.0, stipulating *P*=0.05), we noted that patients having either *TCF12* mutated or *TCF12* loss of heterozygosity (LOH) tended to be associated with shorter survival (Supplementary Fig. 4). To gain further insight into the role of *TCF12* mutation in oligodendroglioma, we sequenced 75 grade II tumours identifying one mutation carrier (P212fs*31; Supplementary Data 1). The observation that the frequency of *TCF12* mutations is higher in AO as compared with grade II tumours (*P*=0.049, *χ*^2^-test) is compatible with *TCF12* participating in the generation of a more aggressive phenotype.

### *TCF12* bHLH mutants compromised transactivation

**Figure 3:**
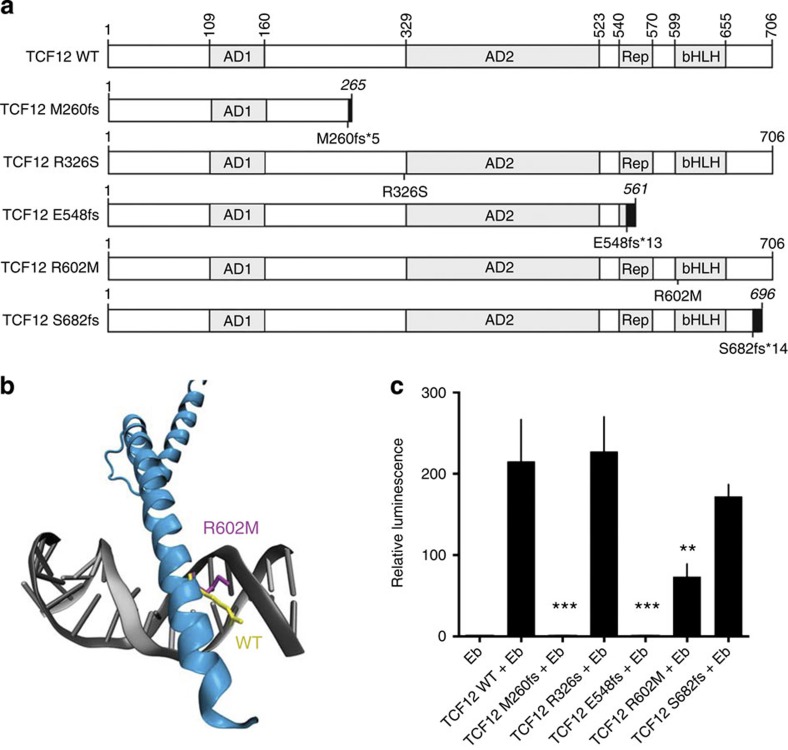
*TCF12* mutations altering the bHLH domain result in impaired transactivation. (**a**) Schematic view of the wild-type and mutant TCF12 proteins for which the transactivation capacity has been assessed. Upper panel: wild-type human TCF12, functional domains in grey—activation domain 1 (AD1), activation domain 2 (AD2), repressor domain (Rep) and bHLH domain (bHLH). Lower panel: resulting truncated proteins. Black boxes indicate non-related amino-acid sequences resulting from frameshift mutations (fs), and truncated proteins size is in italic. (**b**) Schematic structure of the bHLH domain of TCF12 (blue) bound to DNA (grey). WT R602 (yellow) and mutant M602 (purple) residues are indicated. (**c**) E-box-luciferase reporter plasmid (Eb) was transfected alone or in combination with TCF12 wild-type or mutant expression plasmids. Both frameshift mutants that lack the bHLH DNA binding domain completely abolish TCF12 transcriptional activity. All samples were run in triplicate in four independent experiments. Data were normalized to control renilla luciferase. Values are mean±s.d. ***P=0.0002, **P=0.0018 (Student’s *t*-test).

To explore the functional consequences of *TCF12* mutation, we tested the transcriptional activity of several mutants (Fig. 3). We tested the frameshift mutations M260fs*5 and E548fs*13, which in the germline cause coronal craniosynostosis^10^ and S682fs*14, since introduction of a C-terminal premature stop codon may result in escape from non-sense-mediated decay. We also tested the missense mutation R602M, which is predicted to destabilize the bHLH domain required for DNA binding and dimerization (Fig. 3) and whose adjacent residue (R603) has been found recurrently mutated in colon cancer^16^. Finally, we tested the missense mutation R326S, since mutations of adjacent G327 have been reported in lung adenocarcinoma^17^. The frameshift mutants M260fs*5 and E548fs*13 completely abolished TCF12 transactivation, consistent with the lack of bHLH DNA-binding domain (Fig. 3). R602M retained only 34% of WT transcriptional activity (*P*=0.0018, Student’s *t*-test; Fig. 3). We did not observe significant modulation of transactivation for the R326S and S682fs*14 mutants, although the latter consistently showed decreased activity (Fig. 3).

### Downregulation of pathways in *TCF12* bHLH mutants

We profiled gene expression in 8 *TCF12*-mutated and 45 wild-type tumours within 1p/19q co-deleted samples (Supplementary Table 1). *TCF12* mutation was associated with significant enrichment of immune response pathways (Supplementary Data 4). Restricting the analysis to tumours with the *TCF12*-altered bHLH domain (*n*=6), we found downregulation of pathways featuring known partners of TCF12, such as TCF21, EZH2 and BMI1 (ref. 18) (Supplementary Table 2). Interestingly, we found decreased activity of genes sets related to E-cadherin (*CDH1*), which is a TCF12 target gene associated with tumour phenotype^18^. Since the promotor sequences of *CDH1* and *BMI1* feature E-box motifs and are modulated by the bHLH binding^19^,^20^, this provides a mechanistic basis for change in gene expression associated with mutant *TCF12*.

### Mutant TCF12 proteins show subcellular localization changes

**Figure 4:**
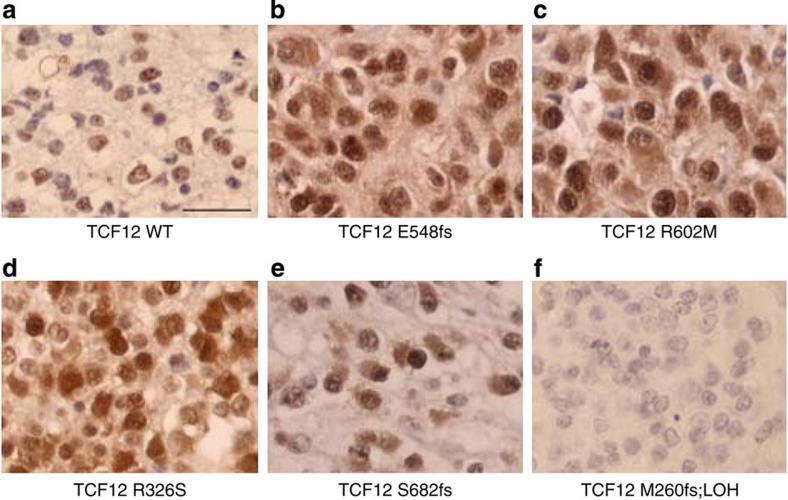
TCF12 is highly expressed in a subset of anaplastic oligodendroglioma. Representative TCF12 immunostainings are shown: (**a**) wild-type TCF12 tumours show nuclear staining in a heterogeneous cell population. (**b**–**e**) Mutant TCF12 tumours show strong nuclear and cytoplasmic staining. (**f**) Mutant M260fs (resulting in a truncated protein) is associated with 15q21.3 LOH and shows no staining. Scale bar, 50 μm.

We evaluated TCF12 expression and subcellular localization for all of our 11 *TCF12*-mutated tumours (10 AO and 1 oligodendroglioma grade II) and 11 *TCF12* wild-type tumours by immunohistochemistry. All *TCF12* wild-type tumours showed nuclear expression in a heterogeneous cell population (Fig. 4; Supplementary Fig. 5), whereas several *TCF12*-mutated tumours showed nuclear and cytoplasmic staining (Fig. 4; Supplementary Fig. 5). Interestingly, mutations abolishing transcriptional activity were associated with increased staining, suggesting inactive mutant protein accumulation.

### TCF12 mutations associate with aggressive tumour phenotype

**Figure 5:**
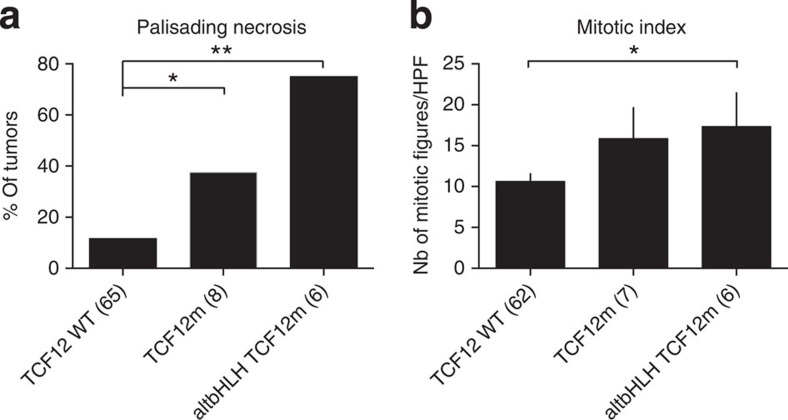
*TCF12* mutation correlates with a higher necrotic and mitotic index. (**a**) Percentage of palisading necrosis in tumours with wild-type *TCF12*, all tumours mutated for *TCF12* or only altered bHLH *TCF12* mutants; *P=0.02, **P=0.004. (**b**) Mitotic index in *TCF12* wild-type, *TCF12*-mutated and altered bHLH *TCF12* mutants; *P=0.039, mean±s.e.m. CN, copy number; LOH, loss of heterozygosity; HPF, high-power field. The number of samples is indicated in parenthesis.

We profiled the extent of necrosis, microvascular proliferation and the mitotic index available for *TCF12* wild-type or mutated tumours. A significant increase in palisading necrosis (Fig. 5) as well as a trend towards a higher mitotic index was associated with *TCF12* mutation, consistent with a more aggressive phenotype (Fig. 5). Intriguingly, tumours harbouring disruptive bHLH domain mutations exhibited the highest proportion of palisading necrosis and mitotic figures.

## Discussion

Our genome sequencing of AO has confirmed the mutually exclusive mutational profile in *IDH*mut-1p/19q co-deleted and *IDH*mut non-1p/19q co-deleted tumour subtypes, which reflect distinct molecular mechanisms of oncogenesis—consistent with the requirement for either 1p/19q co-deletion or *TP53* mutation post *IDH* mutation. Moreover, as previously proposed, the genomic abnormalities in *IDH*mut-1p/19p co-deleted tumours are consistent with one common mechanism of tumour initiation being through 1p/19q loss, mutation of *IDH1* or *IDH2* and *TERT* activation through promoter mutation^2^, which in turn predisposes to deactivation of *CIC*, *FUBP1*, *NOTCH* and activating mutations/amplifications in the PI3K pathway.

We identified and replicated mutations in *TCF12*, a bHLH transcription factor that mediates transcription by forming homo- or heterodimers with other bHLH transcription factors. Tcf12 is highly expressed in neural progenitor cells during neural development^21^ and in cells of the oligodendrocyte lineage^22^.

We found that mutations generating truncated TCF12 lacking the bHLH DNA-binding domain abrogate the transcriptional activity of TCF12. In addition, single residue substitutions such as R602M within the bHLH domain also dramatically reduce TCF12 transcriptional ability. Finally, we found that the loss of TCF12 transcriptional activity was associated with a more aggressive tumour phenotype. Although speculative, our expression data provides evidence that the effects of *TCF12* mutation on AO development may be mediated in part through E-cadherin related pathway. Indeed, this was one of the pathways down-regulated in mutated tumours and intriguingly *CDH1* has been implicated in metastatic behaviour in a number of cancers^18^,^23^. It is likely that some *TCF12* mutations may have subtle effects on bHLH function or act through independent pathways. Irrespective of the downstream effects of *TCF12* mutation on glioma, our data are compatible with *TCF12* having haploinsufficient tumour suppressor function. *TCF12* haploinsufficiency has previously been reported in patients with coronal craniosynostosis and in their unaffected relatives^10^. Strikingly, 3 of the 11 mutations we identified in AO, which concern residues M260, E548 and R602, cause coronal craniosynostosis^10^,^24^. Although speculative, collectively these data raise the possibility that carriers of germline *TCF12* mutations may be at an increased risk of developing AO.

To our knowledge, this study represents the largest sequencing study of AO conducted to date. However, given the number of tumour-normal pairs we have analysed and the mutational frequency in AO, we were only well powered to identify genes that have a high-frequency mutations (that is, >10%). Hence further insights into the biology of AO should be forthcoming through additional sequencing initiatives and meta-analyses of these data.

## Methods

### Patient samples and consent

Samples were obtained with informed and written consent and the study was approved by Comité de Protection des Personnes Ile de France-VI (October 2008) of respective hospitals participating in the Prise en charge des oligodendrogliomes anaplasiques (POLA) network. All patients were aged 18 years or older at diagnosis, and tumour histology was centrally reviewed and validated according to World Health Organization (WHO) guidelines^25^. Exome sequencing was conducted on samples from 51 AO patients (33 male; median age 49 years at diagnosis, range 27–81). For targeted follow-up analyses, we studied the tumours from an additional 83 AO patients and 75 patients with grade II tumours. A summary of each of the tumour cohorts and respective pathological information on the patients is provided in Supplementary Table 1.

### DNA and RNA extraction

Germline DNA was extracted from EDTA-venous blood samples using QIAquick PCR Purification Kits (Qiagen Ltd). Tumour DNA was extracted from snap-frozen tumour samples using the iPrep ChargeSwitchH Forensic Kit, according to manufacturer’s recommendations. DNAs were quantified and qualified using a NanoVue Plus spectrophotometer (GE Healthcare Life Sciences) and gel electrophoresis. RNA was extracted from tumours lysed by Lysing Matrix D tube and FastPrep instrument (MP Biomedicals) using the iPrep Trizol Plus RNA Kit (Life Technologies). Stringent criteria for RNA quality were applied to rule out degradation, specifically a 28S/18S ratio >1.8.

### SNP array analysis

In total, 115 samples from tumours were genotyped using Illumina SNP microarrays: 32 samples with Illumina 370-Duo 1.0 BeadChips, 31 with Human610-Quad, 46 with HumanOmniexpress-12V1 and 6 with HumanCore-12v1. Raw fluorescent signals were imported into BeadStudio software (Illumina) and normalized to obtain log R ratio and B-allele frequency (BAF) values. The tQN normalization procedure was then applied to correct for asymmetry in BAF signals due to bias between the two dyes used in Illumina assays. Genomic profiles were divided into homogeneous segments by applying the circular binary segmentation algorithm to both log R ratio and BAF values. We then used the Genome Alteration Print method to determine the ploidy of each sample, the level of contamination with normal cells and the allele-specific copy number of each segment. Chromosome aberrations were defined using empirically determined thresholds as follows: gain, copy number ≥ploidy+1; loss, copy number ≤ploidy −1; high-level amplification, copy number >ploidy+2; homozygous deletion, copy number=0. Finally, we considered a segment to have undergone LOH when the copy number of the minor allele was equal to 0. Lists of homozygous deletions and focal amplifications, defined by at least five consecutive probes, were generated and verified manually to remove doubtful events. Significantly recurrent copy number changes were identified using the GISTIC2.0 algorithm^26^.

### *TERT* promoter mutation sequencing

Characterized mutations in the *TERT* promoter, C228T and C250T variants with G>A nucleotide substitutions at genomic positions 1,295,228 bp and 1,295,250 bp (hg19), respectively, were obtained by Sanger sequencing. Primer sequences were: TERT-F—5′-GGCCGATTCGACCTCTCT-3′ and TERT-R 5′-AGCACCTCGCGGTAGTGG-3′.

### Whole-exome sequencing

DNA was quantified using the Quant-iT PicoGreen dsDNA Assay Kit (Life Technologies). Libraries were generated robotically using the SureSelectXT Automated Human All Exon Target Enrichment for Illumina Paired-End Multiplexed Sequencing (Agilent) as per the manufacturer’s recommendations. Libraries were quantified using the Quant-iT PicoGreen dsDNA Assay Kit (Life Technologies) and the Kapa Illumina GA with Revised Primers-SYBR Fast Universal kit (D-Mark). Average size of the fragment was determined using a LaChip GX (PerkinElmer) instrument. Sequencing was performed by pooling four libraries per lane at a 9-pM dilution on an Illumina HiSeq 2,000 instrument for 2 × 100 cycles using the recommended manufacturer’s conditions. PhiX control was added at 1% on each lane. BCL2FASTQ (Illumina) was used to convert bcl files to fastqs (v 1.8.4). Coverage statistics are summarized in Supplementary Fig. 1. Paired-end fastq files were extracted using Illumina CASAVA software (v.1.8.1, Illumina) and aligned to build 37 (hg19) of the human reference genome using Stampy and Burrows–Wheeler Aligner^27^, and PCR duplicates were removed with PicardTools 1.5. We assessed coverage of consensus coding sequence bases using Genome Analysis Toolkit^28^ v2.4-9. Somatic single-nucleotide variants were called using MuTect^29^ and the Genome Analysis Toolkit v2.4-9, and indels using IndelGenotyper. We excluded potential Covaris-induced mutations as per Costello *et al*.^30^ using in-house scripts. Confirmation of selected single-nucleotide variants including *TCF12*, *CIC*, *FUBP1*, *SYNE1*, *FAT1*, *SETD2*, *RBPJ*, *NOTCH1*, *IDH1* and *IDH2* was performed by Sanger sequencing implemented on ABI 3,300 × l platforms (Applied Biosystems, Foster City, USA). Primer sequences are detailed in Supplementary Data 5. In all cases, Sanger sequencing was 100% concordant with next-generation sequencing.

We used MutSigCV^8^ version 1.4 to identify genes harbouring more non-synonymous mutations than expected by chance, given gene size, sequence context and the mutation rate. We used as genomic covariates the mean expression level of each gene in our AO expression data set, the DNA replication time and the HiC statistic of chromatin state available in MutSig reference files. To increase our ability to identify cancer drivers and delineate associated oncogenic pathways for AO, we incorporated mutation data from multiple tumour types using Oncodrive-fm^14^ implemented within the IntOGen-mutations platform^15^.

### Transcriptome sequencing

Extracted RNA was cleaned using the RNeasy MinElute Cleanup Kit (Qiagen) and the RNA integrity assessed using an Agilent 2,100 Bioanalyzer and quantified using a Nanodrop 1,000. Libraries for stranded total RNA-seq were prepared using the Illumina Stranded Total RNA protocol (RS-122-2301). Libraries were assessed by the Agilent 2,100 Bioanalyzer. Sequencing was performed by pooling four libraries per lane at a 9-pM dilution on an Illumina HiSeq 2,000 instrument for 2 × 100 cycles using the recommended manufacturer’s conditions. PhiX control was added at 1% on each lane. BCL2FASTQ was used to convert bcl files to fastqs (v 1.8.4). Paired-end reads from RNA-seq were aligned to the following database files using Burrows–Wheeler Aligner 0.5.5: (i) the human GRCh37-lite reference sequence, (ii) RefSeq, (iii) a sequence file representing all possible combinations of non-sequential pairs in RefSeq exons and (iv) the AceView database flat file downloaded from UCSC, representing transcripts constructed from human expressed sequence tag (ESTs). The mapping results from databases (ii)-(iv) were aligned to human reference genome coordinates. The final BAM file was constructed by selecting the best alignment. To identify fusion transcripts, we analysed RNA-seq data using Chimerascan software^31^ (version 0.4.5). As advocated, algorithmic output was analysed for high-confidence fusion transcripts imposing filters: (i) spanning reads >2 (ii) total supported reads ≥10 (ref. 32). In absence of corresponding paired normal tissue samples, we made use of data from the human body map project data to identify fusions seen in normal tissue.

### *TCF12* sequencing in the validation series

PCR amplification of 21 amplicons covering each exon of *TCF12* on DNA extracted from fresh-frozen tumours were performed using Fluidigm technology according to the manufacturer’s recommendations. The 21 PCR products from one tumour sample were then equimolarly pooled and submitted to the MiSeq (Illumina) sequencing as per the manufacturer’s protocol. All mutations were validated by Sanger sequencing. Somatic mutations were confirmed using paired constitutional DNA.

### mRNA expression profiling

Gene expression profiles of 71 samples were analysed using Affymetrix Human Genome U133 Plus 2.0 arrays. All samples were normalized in batches using the RMA algorithm (Bioconductor *affy* package), and probe set intensities were then averaged per gene symbol.

### Identification of significantly mutated pathways

Gene set member lists were retrieved online from MSigDB^33^, GO^34^ and SMD^35^ databases. We searched for gene sets harbouring more damaging mutations than expected by chance. Given the set G of all the genes sequenced with sufficient coverage, the set S of tumour samples (of size *n*) and any gene set P, we calculated the probability of observing a number of mutations equal or greater to that observed in P across the *n* samples according to a binomial law *B*(*k*, *p*), with *k*=*n* × *L*(P) and the mutation rate *p*=*A*(G, S)/(*n* × *L*(*G*)), where *L*(X) is the sum of the lengths (in bp) of all genes/exons from a gene set X, and *A*(G, S) is the total number of mutations observed in all the targeted sequences across all the samples from S.

### Deregulated gene sets in TCF12 mutant samples

We performed a moderate *t*-test using LIMMA R package to identify significantly differentially expressed genes between *TCF12* mutant samples and *TCF12* wild-type samples (*P*<0.05 and absolute log fold change >0.6). Biological pathways and gene set member lists were retrieved online from MSigDB^33^, GO^34^ and SMD^35^ databases. Enrichment *P* values were computed from a hypergeometric test between those gene sets and the initial list of differentially expressed genes. To visualize gene set activity, for each gene set defined as target genes of either *CDH1*, *TCF21*, *BMI1*, *EZH2* and found to be significantly deregulated in *TCF12* bHLH-altered samples compared with *TCF12* wild-type samples in O3 samples with co-deletion, we retrieved the complete member list from MSigDB^33^ and computed a global mean gene expression value in each sample. We then ranked the samples according to the later global mean expression value for each of these gene sets.

### Structure modelling

The Swiss Model^36^ server was used to model mutated TCF12 and VMD software^37^ used to align the structures of wild-type and mutated TCF12 proteins with STAMP (STructural Alignment of Multiple Proteins)^38^. Prediction of the functional effect of the R602M mutation on TCF12 was made using Project HOPE^39^.

### Statistical analysis

Statistical analysis was carried out using R3.0.1 software. A *P* value ≤0.05 was considered to be significant. Continuous variables were analysed using the Student’s *t*-test or Mann–Whitney test. Categorical data were compared using Fisher’s exact test or the *χ*^2^-test. Overall survival of patients was the end point of the analysis. Survival time was calculated from the date of tumour diagnosis to the date of death. Patients who were not deceased were censored at the date of last contact. Mean follow-up time was computed among censored observations only. Kaplan–Meier survival curves according to genotype were generated and the homogeneity of the survival curves between genotypes was evaluated using the log-rank test. Power to demonstrate a relationship between mutation status and overall survival was estimated using sample size formulae for comparative binomial trials^40^.

### Cell culture

Human embryonic kidney HEK293T cell line (American Type Culture Collection) was maintained in a 5% CO_2_-regulated incubator in DMEM Glutamax (Life Technologies), completed with 10% fetal bovine serum and penicillin/streptomycin (Life Technologies).

### Plasmid construction

To construct the TCF12 wild-type plasmid, we cloned, by Gateway recombination (Life Technologies), a pENTR221 TCF12 Ultimate ORF Clone (Life Technologies) into a pDEST12 lentiviral vector (kind gift from P. Ravassard), under the control of hCMV promoter. The M260fs*5 and R326S mutations were generated by PCR mutagenesis using the Q5 Site-directed Mutagenesis kit (New England Biolabs) on pENTR221 TCF12 plasmid (primer sequences are detailed in Supplementary Data 5) and then cloned into the pDEST12 vector by LR Gateway cloning. Synthetic NdeI/MfeI fragments (encompassing sequences from exon 16 to the TAG stop codon of the ENST00000438423 isoform), containing the mutations E548fs*13, R602M and S683fs*14, were obtained from GeneCust, then substituted into pENTR221 and finally cloned by Gateway recombination into the pDEST12 plasmid. All expression plasmids were sequenced before use.

### Luciferase expression assays

For each experiment, 10^5^ exponentially growing HEK293T cells were seeded in 12-well plates and transfected 24 h later using Fugene6 (Promega), according to manufacturer’s instructions, with 0.3 μg of a reporter plasmid encoding firefly luciferase under the control of an E-box-responsive element (Eb, kind gift from A. Lasorella), or 0.3 μg of Eb plasmid and 0.7 μg of a *TCF12* wild-type expression plasmid, or 0.3 μg of Eb plasmid and 0.7 μg of either *TCF12* mutant (M260fs*5, R326S, E548fs*13, R602M or S628fs*14) expression plasmid. For all points, data were normalized by adding 30 ng of renilla luciferase expression plasmid (pGL4.73, Promega, gift from F. Toledo). Cells were harvested 24 h after transfection, and luminescence was monitored using the Dual-Glo Luciferase assay system (Promega), according to the manufacturer’s instructions, on a Spectramax M4 instrument and SoftMax Pro 6.2.2 software. All samples were run in triplicate, in four independent experiments.

### Immunohistochemistry

Paraffin-embedded tumour sections were deparaffinized using standard protocols. Heat-mediated antigen retrieval was achieved by boiling sections in a pressure cooker with Citrate buffer at pH 6. Sections were blocked in 10% goat serum in PBS+0.5% Triton X-100 for 30 min prior to incubation with an anti-TCF12 antibody (Proteintech Cat no.: 14419-1-AP) and then revealed using the Polink-2 HRP Plus Rabbit DAB Detection System (GBI Labs:D39-6). Photographs were taken at × 400 magnification and processed using AxioVision software (Zeiss). The mitotic index in tumours was recorded as the number of mitotic figures in 10 high-power fields.

### TCGA data

To complement our analysis, we made use of exome sequencing data on AO tumours generated by the TCGA (Supplementary Data 2).

## Additional information

**Accession codes:** All whole-exome sequencing and transcriptome data have been deposited at the European Genome-phenome Archive (EGA), which is hosted by the European Bioinformatics Institute (EBI), under the accession code EGAS00001001209. mRNA expression and SNP data can be accessed through ArrayExpress under accession numbers E-MTAB-2768 for mRNA expression data, and E-MTAB-3457, E-MTAB-3458, E-MTAB-2772 and E-MTAB-2771 for SNP data.

**How to cite this article:** Labreche, K. *et al*. *TCF12* is mutated in anaplastic oligodendroglioma. *Nat. Commun.* 6:7207 doi: 10.1038/ncomms8207 (2015).

## Supplementary Material

Supplementary Information Supplementary Figures 1-5, Supplementary Tables 1-2

Supplementary Data 1 Genomic and histological characteristics of tumors analysed

Supplementary Data 2 Significantly mutated genes in anaplastic oligodendroglioma in this series and in TCGA samples

Supplementary Data 3 Significantly mutated gene sets. (a) Gene sets harboring significantly more mutations than expected by chance are indicated. (b) Gene sets highlighted in the study, with detailed number of mutations among the main mutated genes of each set (identified as significantly mutated through MutSigCV or as significantly biased through Oncodrive-fm).

Supplementary Data 4 List of pathways and gene sets significantly enriched in genes significantly deregulated between TCF12 mutant samples and TCF12 wt samples. Gene set member lists were retrieved online from MSigDB, GO and SMD databases and hypergeometric tests were performed between each gene set and the list of significantly deregulated genes.

Supplementary Data 5 Primer sequences
