# Supplementary material for: TCF12 is mutated in anaplastic oligodendroglioma
Source: Nat Commun. 2015 Jun 12;6:7207. doi: 10.1038/ncomms8207 (PMC4490400; doi:10.1038/ncomms8207)
Supplement: Supplementary Information — Supplementary Figures 1-5, Supplementary Tables 1-2 [file ncomms8207-s1.pdf]

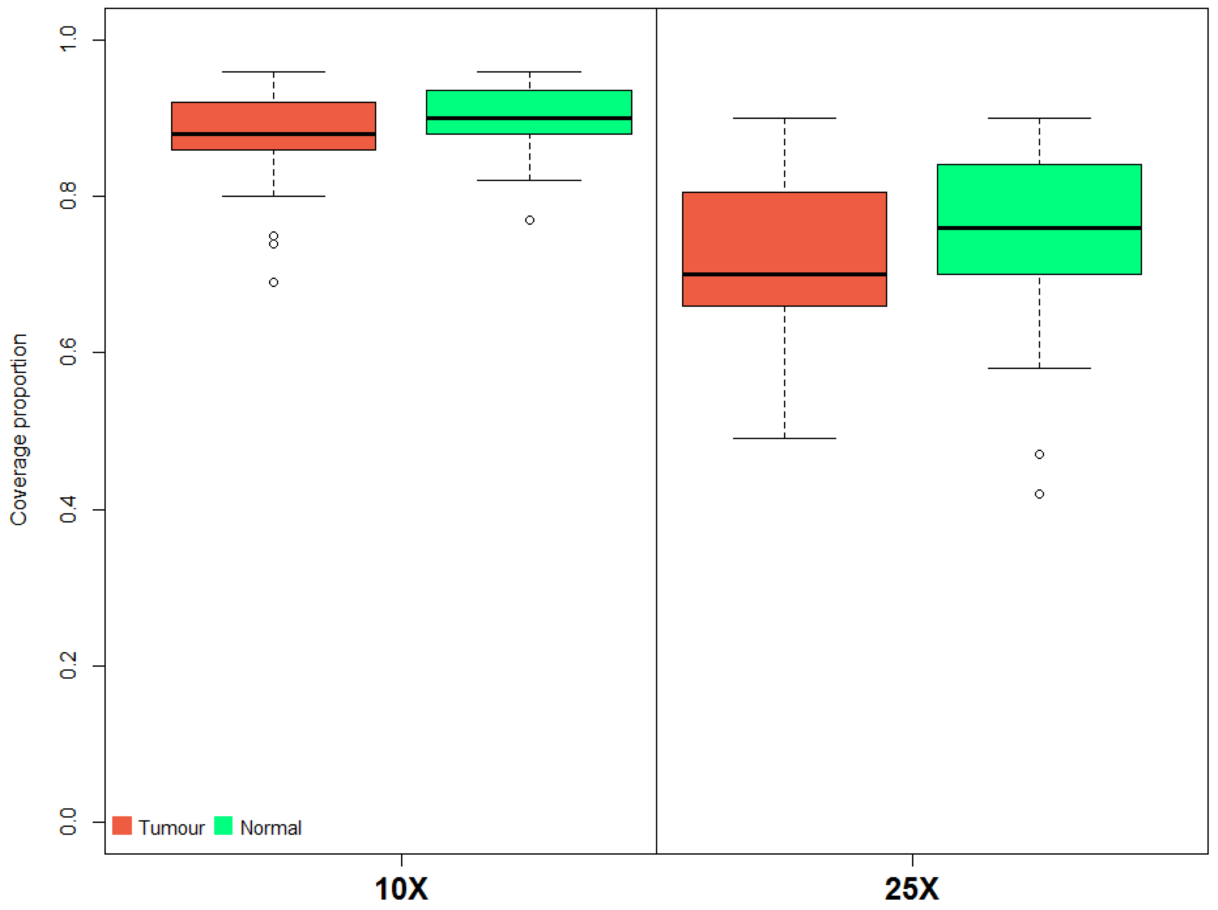

Supplementary Figure 1: Coverage of exome sequencing Proportion of bases in targeted exons sequenced at a depth of 10× and 25× for 51 AOs tumours and their normal counterparts. Boxes divided by median values. Length of boxes corresponds to interquartile range and whiskers correspond to 1.5 interquartile ranges.

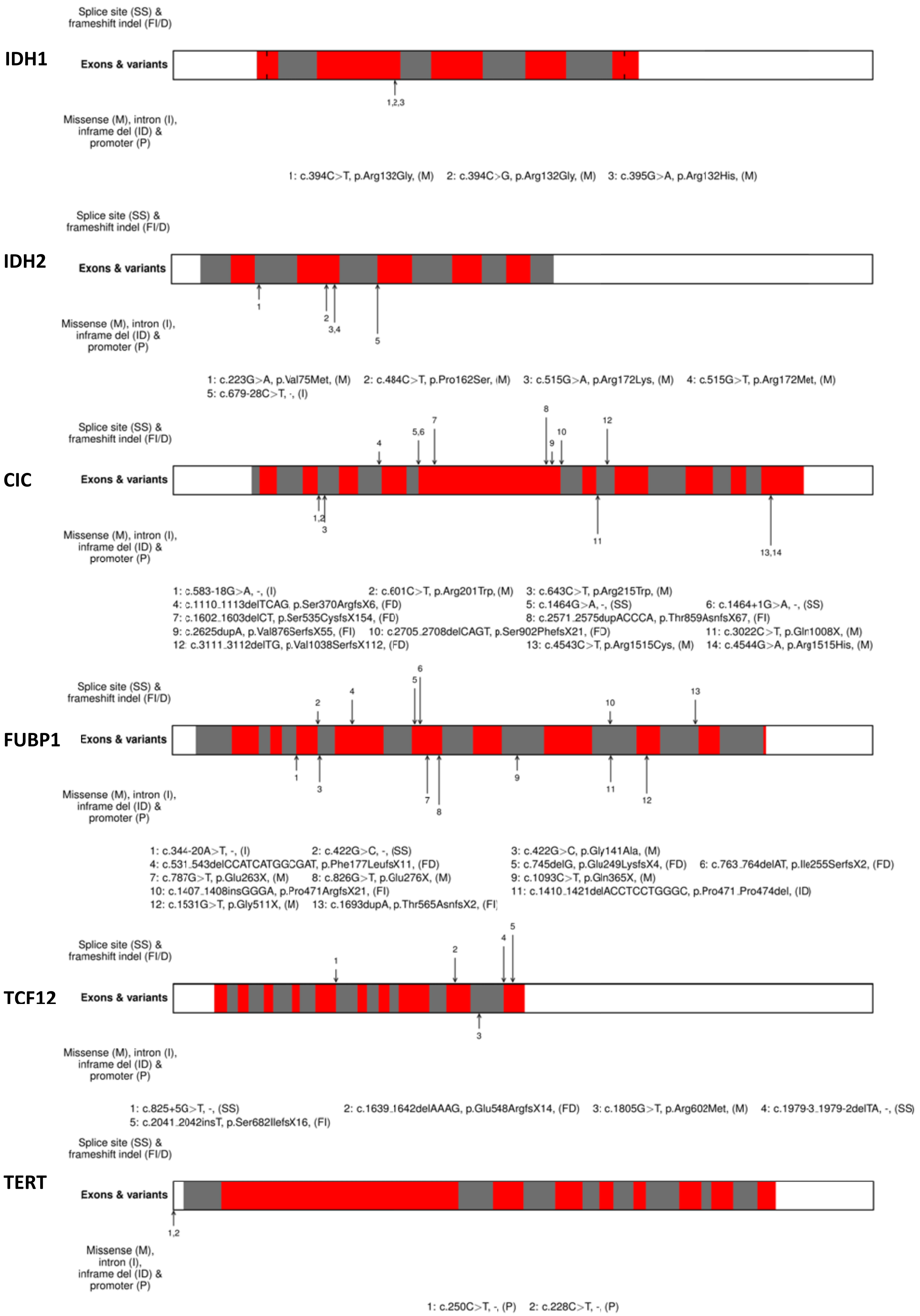

Supplementary Figure 2: Location of mutations in key genes recurrently mutated in AO Transcripts are plotted 5' to 3'; untranslated regions are not colored; coding regions of exons are shown in alternating red and gray. The variants track shows the distribution of mutations.

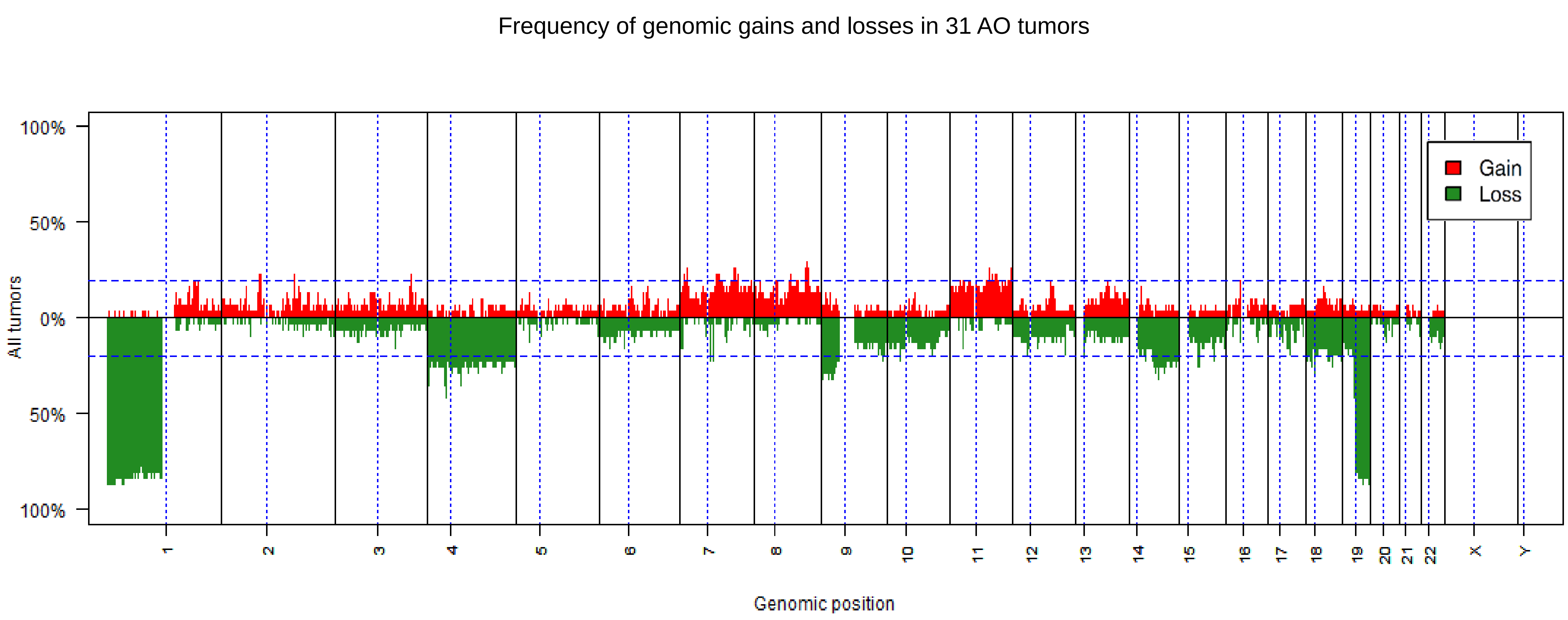

Supplementary Figure 3: Frequency of genomic gains and losses in 31 AO samples. Vertical solid lines separate chromosomes, and vertical dashed lines indicate centromeres positions. Gains and losses frequency peaks were computed for each genomic position targeted by SNP arrays (excluding sexual chromosomes and positions within known frequent germline CNVs).

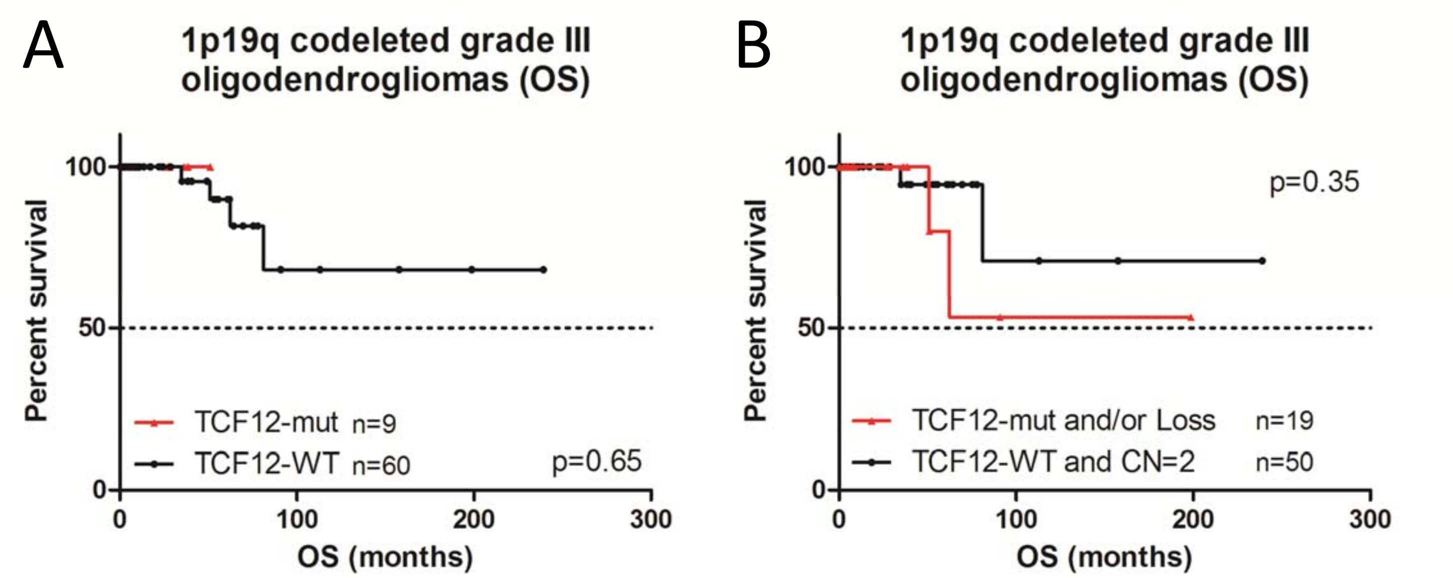

Supplementary Figure 4: Overall survival from of 1p/19q co-deleted anaplastic oligodendrogliomas according to TCF12 mutation status. Overall survival analysis of (a) TCF12 mutant (red line) and TCF12 wild-type glioma patients (black line), (b) TCF12 mutant ± TCF12 loss of heterozygosity (LOH; red line) and TCF12 wild-type patients without any copy number change (black line). The median follow-up was 35 months. Log-rank (Mantel-Cox) test was used to evaluate the significance of differences.

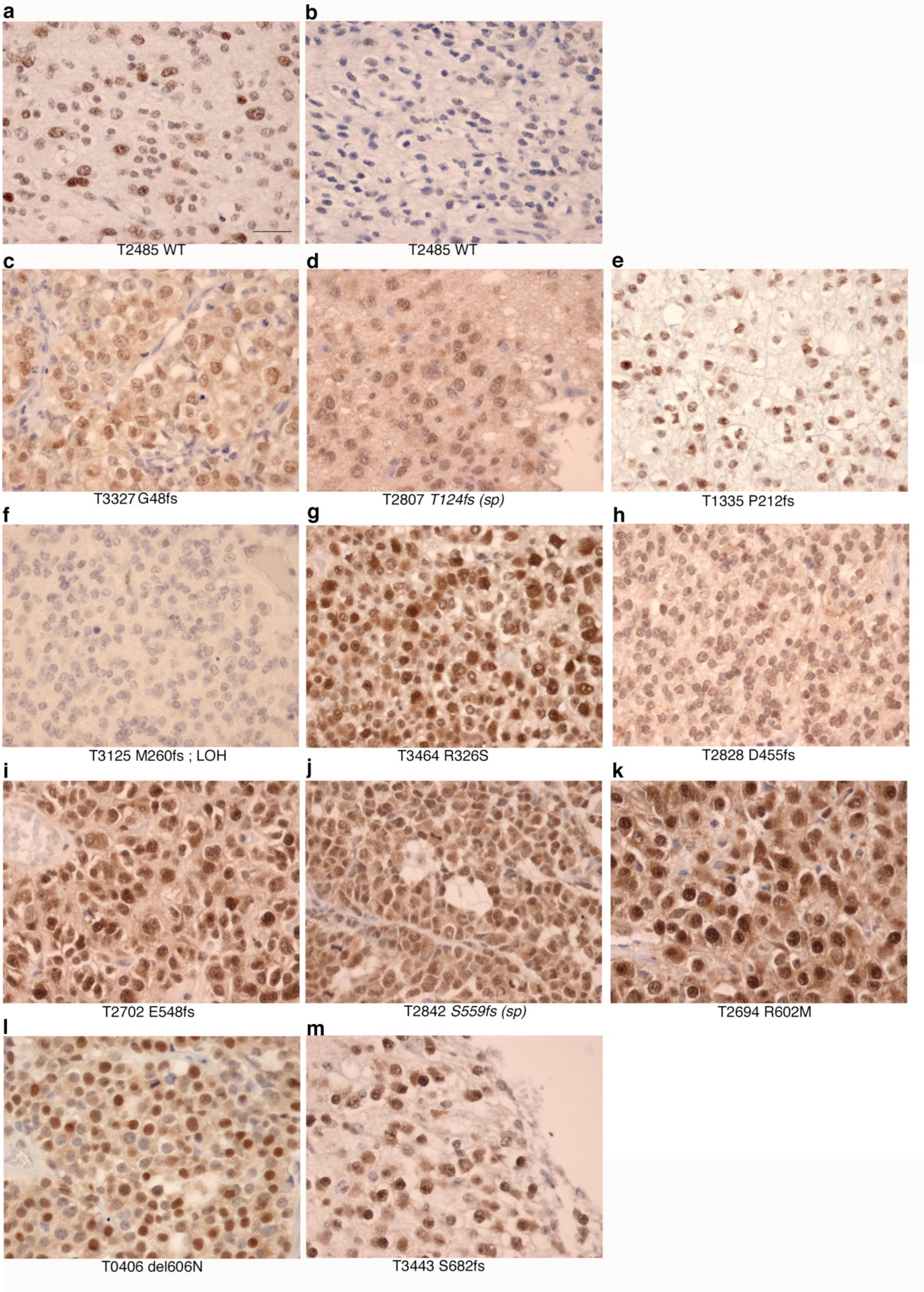

Supplementary Figure 5: TCF12 protein expression in anaplastic oligodendroglioma . (a-m) TCF12 immunostaining on paraffin sections of 1p/19q co-deleted AO. (a) Representative IHC of wild type TCF12 tumor shows nuclear staining in a heterogeneous cell population, the scale bar corresponds to 5u (b) TCF12 negative field from the same tumor, (c-e,h) N-terminal heterozygous frame shift (fs) mutants show reduced positive staining, corresponding only to the residual wild type allele, (f) N-terminal frame shift mutant M260fs with loss of heterozygosity at 15q21.3 stains negative, (g,i-k,m) C-terminal TCF12 mutants show a characteristic strong nuclear and cytoplasmic staining. The in-frame deletion in (l) showing only nuclear staining is the exception.

Arm	# Genes	Amp frequency	Amp z-score	Amp q-value	Del Frequency	Del z-score	Del q-value
1p	2121	0	-0.689	0.951	0.84	14.9	0
1q	1955	0.08	-0.177	0.951	0.17	1.64	0.194
2p	924	0.13	0.84	0.712	0	-1.6	0.951
2q	1556	0.13	0.838	0.712	0	-1.6	0.951
3p	1062	0.03	-0.998	0.951	0.07	-0.388	0.951
3q	1139	0.03	-0.998	0.951	0.07	-0.388	0.951
4p	489	0	-1.44	0.951	0.29	4.04	0.000215
4q	1049	0	-1.44	0.951	0.29	4.03	0.000215
5p	270	0.1	0.204	0.951	0	-1.63	0.951
5q	1427	0.06	-0.438	0.951	0	-1.66	0.951
6p	1173	0	-1.66	0.951	0.06	-0.437	0.951
6q	839	0	-1.66	0.951	0.06	-0.437	0.951
7p	641	0.16	1.48	0.453	0	-1.57	0.951
7q	1277	0.16	1.48	0.453	0	-1.57	0.951
8p	580	0.16	1.48	0.453	0	-1.57	0.951
8q	859	0.16	1.48	0.453	0	-1.57	0.951
9p	422	0.09	0.00747	0.951	0.28	3.63	0.000932
9q	1113	0.08	-0.057	0.951	0.24	2.96	0.00848
10p	409	0	-1.57	0.951	0.16	1.48	0.194
10q	1268	0	-1.57	0.951	0.16	1.48	0.194
11p	862	0.23	2.76	0.0574	0	-1.51	0.951
11q	1515	0.23	2.75	0.0574	0	-1.51	0.951
12p	575	0	-1.63	0.951	0.1	0.203	0.82
12q	1447	0	-1.63	0.951	0.1	0.2	0.82
13q	654	0.14	1.06	0.712	0.11	0.454	0.745
14q	1341	0	-1.54	0.951	0.19	2.12	0.0744
15q	1355	0.04	-0.873	0.951	0.17	1.56	0.194
16p	872	0.14	0.983	0.712	0.07	-0.231	0.951
16q	702	0.1	0.262	0.951	0.04	-0.957	0.951
17p	683	0.07	-0.231	0.951	0.14	0.984	0.398
17q	1592	0.07	-0.233	0.951	0.14	0.981	0.398
18p	143	0.04	-0.781	0.951	0.23	2.86	0.0103
18q	446	0.04	-0.872	0.951	0.17	1.56	0.194
19p	995	0.05	-0.686	0.951	0.3	4.16	0.00021
19q	1709	0.2	0.901	0.712	0.87	15.2	0
20p	355	0.06	-0.436	0.951	0	-1.66	0.951
20q	753	0.1	0.202	0.951	0	-1.63	0.951
21q	509	0.06	-0.436	0.951	0	-1.66	0.951
22q	921	0.04	-0.957	0.951	0.1	0.261	0.82

Significant Amplification

Significant Deletion

Supplementary Table 1. Significantly recurrent broad copy number changes identified by GISTIC2.0 analysis

a

Rank	Gene Set	p-value
43	ONDER_CDH1_TARGETS_2_UP	1,23E-08
138	CUI_TCF21_TARGETS_2_DN	2,61E-06
443	NUYTTEN_EZH2_TARGETS_UP	3,57E-04
1418	CUI_TCF21_TARGETS_2_UP	1,13E-02
1594	WIEDERSCHAIN_TARGETS_OF_BMI1_AND_PCGF2	1,57E-02
2060	ONDER_CDH1_TARGETS_1_UP	2,91E-02
2225	BMI1_DN_MEL18_DN.V1_DN	3,38E-02

b

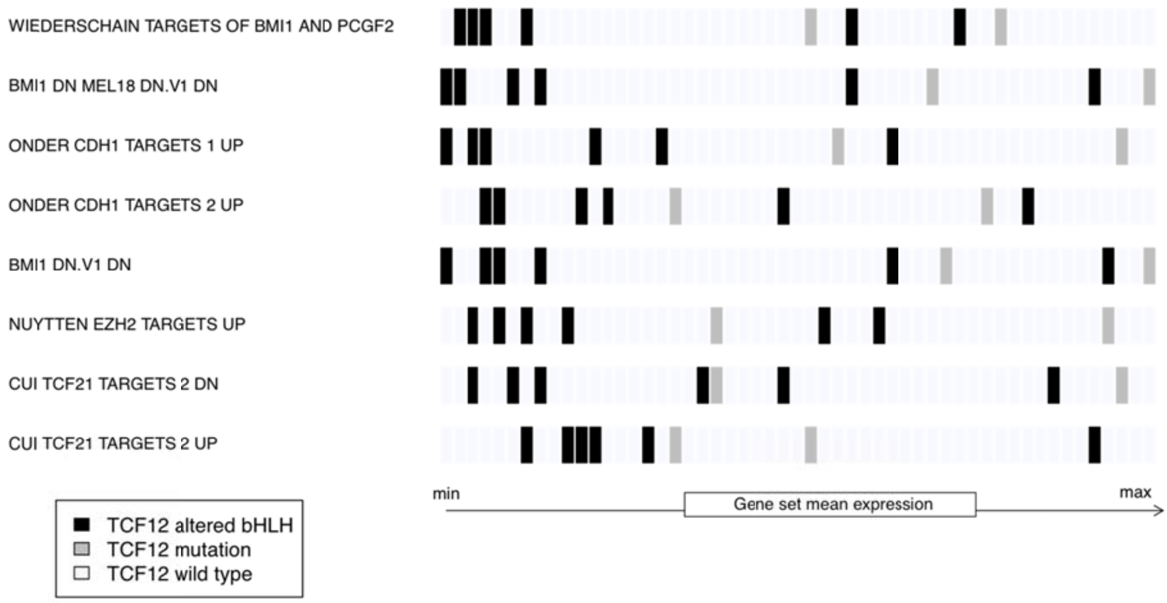

Supplementary Table 2 : **Downregulation of pathways regulated by TCF12 partners in tumors with altbHLH TCF12m mutants**. (a) Target gene sets of CDH1, TCF21, EZH2 and BMI1 are significantly enriched in differentially expressed genes between TCF12 bHLH altered samples and TCF12 wild type tumors. Gene set ranks refer to the p-value ranks among the 19591 gene sets that were tested. CDH1, TCF21, EZH2 and BMI1 target gene set members were retrieved from MSigDB (see ref and methods). (b) Visualisation of samples ranked according to their value of mean gene expression for each gene set. Each row corresponds to the gene set listed on the left, and each rectangle corresponds to a tumor with a color indicating its TCF12 status (wt, altbHLH mutant, or other mutations). Samples with the lowest global expression of all the target genes (whether or not they were initially found differentially expressed in TCF12 bHLH altered samples) are on the left hand side. Reciprocally, samples with the highest global expression of all the target genes are on the right hand side.
